# Cavernous sinus syndrome from clival metastasis as the initial presentation of disseminated prostate adenocarcinoma: a case report and literature review

**DOI:** 10.3389/fonc.2026.1872525

**Published:** 2026-07-08

**Authors:** Abhimanyu Chawla, Sunil Kumar, Sourya Acharya, Shravani Jawadi

**Affiliations:** 1 General Medicine, Datta Meghe Institute of Higher Education and Research Deemed to be University, Wardha, India; 2 General Surgery, Datta Meghe Institute of Higher Education and Research Deemed to be University, Wardha, India

**Keywords:** cavernous sinus syndrome, clival metastasis, inappropriate ADH syndrome, neoplasm metastasis, ophthalmoplegia, prostate-specific antigen, prostatic neoplasms, skull base neoplasms

## Abstract

Prostate adenocarcinoma commonly metastasises to the axial skeleton, but skull base deposits with acute cranial nerve palsies as the first clinical sign are unusual. We report a 68-year-old man admitted with one month history of dyspnoea and weakness, who was found to have severe microcytic anaemia (haemoglobin 6.5 g/dL) with stool positive for occult blood; upper gastrointestinal endoscopy showed only mild antral gastritis. During the first week he developed left-sided ptosis, progressing over three days to complete ophthalmoplegia with binocular diplopia. Gadolinium-enhanced MRI of the brain showed a destructive clival deposit with heterogeneous enhancement extending into the left cavernous sinus. On directed questioning the patient admitted to a five-month history of lower urinary tract symptoms (LUTS) dismissed as age-related. Serum prostate-specific antigen was 754 ng/mL. Cross-sectional imaging and multiparametric prostate MRI confirmed a Prostate Imaging Reporting and Data System (PIRADS) 5 lesion with widespread pulmonary, mediastinal nodal, pelvic nodal and skeletal metastases. The hospital course was complicated by severe hyponatraemia with a high urine sodium (43 mEq/L), supporting the syndrome of inappropriate antidiuresis. Transrectal ultrasound-guided biopsy confirmed prostatic adenocarcinoma. The case is a reminder that cranial nerve palsies in an elderly man can be the first visible sign of silent disseminated prostate cancer, and that asking directly about urinary symptoms at every clinical encounter matter, even though such symptoms are not themselves a reliable indicator of early disease.

## Introduction

Prostate cancer remains the second most commonly diagnosed cancer in men worldwide, with global incidence rising from 1.41 million new cases in 2020 to 1.47 million in 2022 (1, 2). Diagnosis is frequently delayed in low- and middle-income settings. In India, 43% of prostate cancers are detected at the distant metastatic stage compared with 8% in the United States (3). The 2024 Lancet Commission projects a doubling of global cases from 1.4 million in 2020 to 2.9 million by 2040, with the largest absolute increases in low- and middle-income countries driven by demographic ageing (4).

Skeletal dissemination is the predominant pattern of metastatic spread, and bone involvement is an independent predictor of mortality in large registry analyses (5). Axial sites, including the vertebrae, ribs and pelvis, are preferentially affected because of drainage through the valveless vertebral venous plexus (6). Skull base deposits are uncommon, estimated at under 0.02% of all intracranial tumours; when present, prostate primary accounts for around one in five cases, and most patients first come to attention with an isolated sixth nerve palsy from clival or cavernous sinus involvement (7).

Cavernous sinus syndrome is defined by involvement of two or more of the structures that traverse the sinus: cranial nerves III, IV, V1, V2 and VI, together with the sympathetic plexus around the internal carotid artery. The syndrome produces ipsilateral ophthalmoplegia, ptosis and pupillary changes. An acute temporal profile over days tends to reflect rapidly growing tumour or inflammation rather than a slowly expanding mass (8).

We report a patient whose initial hospitalisation for anaemia became the stage on which an acute cavernous sinus syndrome revealed widely disseminated prostate adenocarcinoma, despite a five-month history of urinary symptoms the patient had never reported.

## Case presentation

A 68-year-old man presented to the medicine out-patient department with one month of progressive breathlessness on exertion and generalised weakness. His background included hypertension controlled on amlodipine 5 mg once daily.

On admission he was conscious, oriented, and haemodynamically stable (blood pressure 120/80 mmHg, pulse 88/min, respiratory rate 16/min). He was markedly pale. Systemic examination was otherwise unremarkable.

Haemoglobin was 6.5 g/dL (reference range 13.0–17.0 g/dL) with a microcytic hypochromic smear (MCV 69.9 fL; reference range 80–100 fL). One unit of packed red cells was transfused. Stool was positive for occult blood, and an upper gastrointestinal endoscopy was therefore pursued, which showed only mild antral gastritis with no obvious bleeding lesion. Iron studies were not in keeping with simple iron deficiency. Serum ferritin was 329 ng/mL (reference range 17.9–464 ng/mL), iron 166 µg/dL (reference range 49–181 µg/dL), and total iron-binding capacity 306 µg/dL (reference range 250–450 µg/dL), a pattern more suggestive of anaemia of chronic disease. Serum alkaline phosphatase rose from 226 to 276 U/L (reference range 38–126 U/L) over the first week. A serum sodium of 112 mEq/L (reference range 137–145 mEq/L) with a spot urine sodium of 43 mEq/L confirmed continued urinary sodium excretion despite low serum values, a pattern consistent with the syndrome of inappropriate antidiuresis (SIAD).

On the third morning round a subtle ptosis of the left upper eyelid was noted. Over the next three days this progressed to complete ophthalmoplegia of the left eye with binocular diplopia and a sluggishly reactive, mid-position pupil (**Figure 1**). Right eye movements were unaffected. The combination of ipsilateral third, fourth and sixth nerve involvement, ptosis and a pupil with both parasympathetic and sympathetic fibre compromise localised the lesion to the left cavernous sinus.

**Figure 1:**
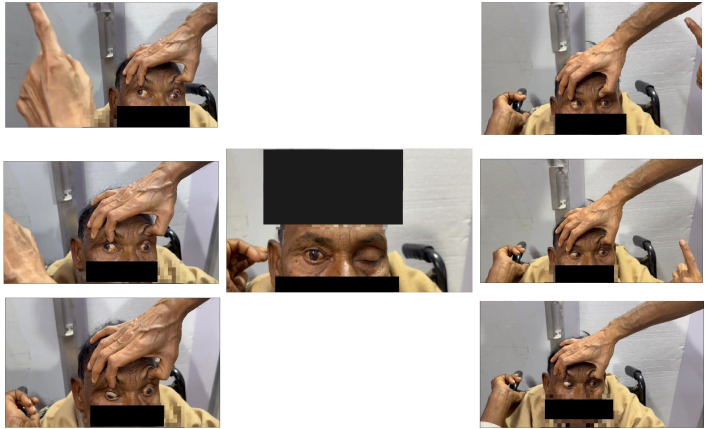
Clinical photograph of the patient demonstrating left-sided ptosis with complete ophthalmoplegia of the left eye. The right eye movements were preserved.

Gadolinium-enhanced magnetic resonance imaging of the brain showed a destructive deposit of the clivus with heterogeneous enhancement and extension into the left cavernous sinus (**Figure 2**).

**Figure 2:**
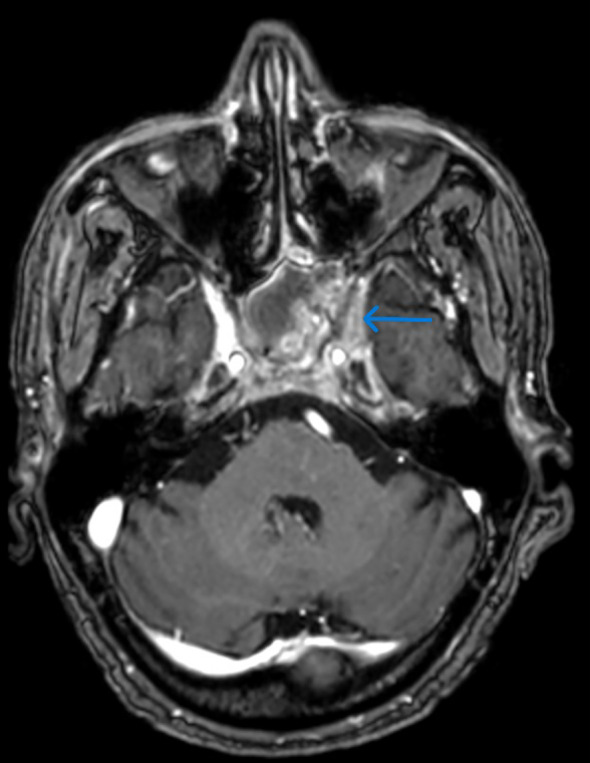
Gadolinium-enhanced T1-weighted magnetic resonance imaging of the brain. A destructive clival deposit with heterogeneous enhancement is visible, with extension into the left cavernous sinus. An axial scroll-through of this sequence is provided as **Supplementary Video 1**.

On directed questioning the patient conceded five months of poor urinary stream and hesitancy, along with 4 kg of unintentional weight loss over two months. He had attributed these symptoms to ageing and had not sought medical attention. Serum prostate-specific antigen was 754 ng/mL (reference range <4 ng/mL).

Neurosurgery was consulted regarding the clival deposit; given the location and complexity of the lesion, a conservative approach was agreed, and the patient was started on intravenous dexamethasone 16 mg/day in divided doses for the cranial nerve deficits. His ophthalmoplegia did not improve.

Contrast-enhanced computed tomography of the thorax, abdomen and pelvis showed multiple bilateral pulmonary nodules consistent with metastases, necrotic mediastinal lymphadenopathy, a necrotic right external iliac node, and diffuse skeletal metastases. A large enhancing soft-tissue component infiltrated the right peri-pelvic and femoral musculature. Multiparametric magnetic resonance imaging of the prostate showed a heterogeneously enhancing mass occupying the central and transition zones with extraprostatic extension and pelvic muscular infiltration, classified as Prostate Imaging Reporting and Data System (PIRADS) 5 (**Figure 3**). Transrectal ultrasound-guided prostatic biopsy confirmed adenocarcinoma (**Figure 4**). The chronological sequence of clinical events, investigations and management is summarised in **Table 1**.

**Figure 3:**
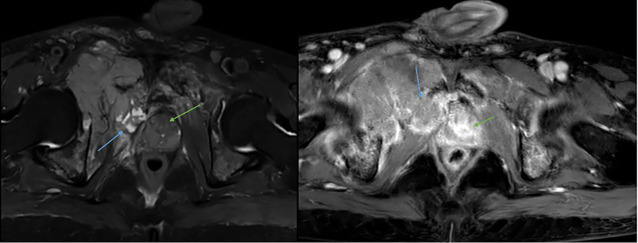
Multiparametric magnetic resonance imaging of the prostate: T2-weighted and axial post-contrast T1-weighted sequences. A heterogeneously enhancing mass occupies the central and transition zones of the prostate (green arrow), with a large right-sided soft-tissue extension (blue arrow).

**Figure 4:**
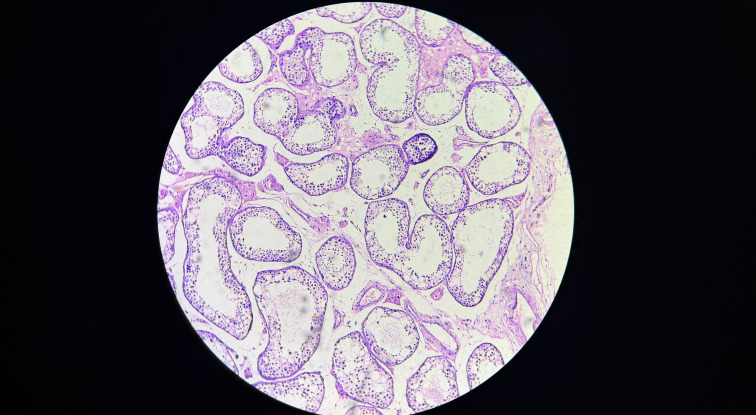
Histopathology of the transrectal ultrasound-guided prostatic biopsy (haematoxylin and eosin stain, ×10 magnification) showing closely packed fused glands and cribriform structures infiltrating fibromuscular stroma with irregular glandular outlines, consistent with adenocarcinoma of the prostate (Gleason score 4 + 5 = 9, Grade Group 5).

**Table 1:** Timeline of clinical events, investigations and management.

Time point	Event
5 months before admission	Onset of lower urinary tract symptoms (poor stream and hesitancy); attributed to ageing and not reported.
2 months before admission	Unintentional weight loss of approximately 4 kg.
1 month before admission	Onset of progressive exertional dyspnoea and generalised weakness.
Day 1 of admission	Hospitalised. Haemoglobin 6.5 g/dL with microcytic hypochromic indices (MCV 69.9 fL); one unit of packed red cells transfused.
Day 2 of admission	Stool positive for occult blood. Upper gastrointestinal endoscopy showed only mild antral gastritis with no bleeding lesion. Iron studies in keeping with anaemia of chronic disease.
Day 3 of admission	Subtle ptosis of the left upper eyelid noted on routine round.
Days 4–6 of admission	Progressive paralysis of left extraocular movements; complete ophthalmoplegia with binocular diplopia. Gadolinium-enhanced MRI of brain demonstrated a destructive clival deposit with extension into the left cavernous sinus. Neurosurgery consulted; conservative approach agreed given lesion complexity. Dexamethasone given for cranial nerve deficits without improvement.
Day 6 of admission	Serum prostate-specific antigen 754 ng/mL. Five-month history of LUTS elicited on directed questioning. Serum sodium nadir 112 mEq/L; spot urine sodium 43 mEq/L. SIAD diagnosed; tolvaptan 30 mg once daily commenced.
Day 7 of admission	Contrast-enhanced computed tomography of thorax, abdomen and pelvis showed bilateral pulmonary metastases, necrotic mediastinal and pelvic lymphadenopathy, and disseminated skeletal metastases.
Day 8 of admission	Multiparametric MRI of prostate: PIRADS 5 lesion in the central/transition zone with extraprostatic extension and pelvic muscular infiltration.
Day 9 of admission	Transrectal ultrasound-guided prostate biopsy performed.
Day 15 of admission	Histopathology confirmed adenocarcinoma of the prostate. Serum sodium corrected to 127 mEq/L.
Day 16 of admission	Advised palliative androgen deprivation therapy; patient apprehensive and preferred alternative medicine. Discharged and lost to subsequent follow-up.

SIAD, syndrome of inappropriate antidiuresis; LUTS, lower urinary tract symptoms; MCV, mean corpuscular volume; MRI, magnetic resonance imaging; PIRADS, Prostate Imaging Reporting and Data System.

Hyponatraemia was corrected with fluid restriction and tolvaptan 30 mg daily, with a rise in serum sodium to 127 mEq/L. After counselling about the prognosis, he was advised palliative androgen deprivation therapy under medical oncology. He remained apprehensive about further treatment and expressed a preference for alternative medicine, which we did not provide. He was discharged and was subsequently lost to follow-up.

## Discussion

Prostate adenocarcinoma presenting with acute cavernous sinus syndrome is an unusual clinical picture, and easy to miss when the patient is already being worked up for a different problem, in this case anaemia. The sixth cranial nerve is most commonly involved because of its long intracranial course along the clivus before it enters the cavernous sinus; case reports over three decades describe isolated abducens palsy as the first sign of an underlying prostate primary (9–11).

Published reports help place our patient in context (**Table 2**). Most describe an isolated sixth nerve palsy (9–12); our patient instead had the third, fourth and sixth nerves involved together with both sympathetic and parasympathetic pupillary signs, which localises the deposit to the cavernous sinus itself rather than the clivus alone. The reported cases also differ in whether the prostate cancer was already known. Yasumizu described three men with castration-resistant disease who developed skull base cranial nerve palsies after the primary was known (13), Lodhia an 83-year-old survivor whose isolated sixth nerve palsy proved to be a new clival metastasis (14), and Satoh a man already on androgen deprivation therapy who developed Collet-Sicard syndrome (cranial nerves IX to XII) from a jugular foramen deposit, the deficit tracking the deposit’s location (15). Yip reported a solitary skull base deposit found only on 68Ga-Prostate-Specific Membrane Antigen Positron Emission Tomography/Computed Tomography (PSMA PET/CT) during staging for Gleason 7 disease, the kind of lesion plain imaging misses (16). In contrast, our patient, like those of O’Boyle and Malloy, presented with the cranial nerve palsy first and the prostate diagnosis second (9, 10); Douglas’s case sits between these groups, a treated prostate cancer not previously known to have spread (11). Dekker’s review of 56 clival metastases from all primaries found prostate, kidney and liver to be the commonest sources, an abducens palsy or diplopia the usual presentation, and the clival deposit preceding the primary diagnosis in 16 cases (12).

**Table 2:** Comparison of the present case with published reports of prostate cancer metastatic to the skull base/clivus.

Report	Age	Primary known before neurological presentation?	Cranial nerves involved	Distinguishing feature
Present case	68	No	III, IV, VI + sympathetic (cavernous sinus syndrome)	Undiagnosed primary; concurrent severe anaemia and SIAD
Douglas 2020 (11)	Not stated	Yes, but metastasis unknown	VI (isolated)	Treated prostate cancer not previously known to be metastatic
O’Boyle 1992 (9)	71	No	VI (isolated)	Diplopia and abducens palsy; biopsy and workup revealed prostate primary
Malloy 2007 (10)	66	No	VI (clivus to cavernous sinus)	Abduction deficit; imaging found previously unknown prostate primary
Yasumizu 2021 (13)	50, 53, 64	Yes (CRPC)	Skull base nerves; ptosis prominent	Three men, all with known castration-resistant disease
Lodhia 2017 (14)	83	Yes (survivor)	VI (isolated)	Long-term prostate cancer survivor; new clival metastasis
Yip 2021 (16)	74	Yes (staging)	None (asymptomatic)	Solitary skull base deposit found on 68Ga-PSMA PET/CT
Satoh 2000 (15)	55	Yes	IX, X, XI, XII (Collet-Sicard)	Jugular foramen deposit; different nerve cluster
Dekker 2017 review (12)	mean 58	Not known in 16 cases	VI most common	Pooled review of 56 clival metastases (various primary sites)

CRPC, castration-resistant prostate cancer; SIAD, syndrome of inappropriate antidiuresis; PSMA, prostate-specific membrane antigen. The present case is highlighted.

Carretta’s 2024 series, which included a PRISMA review of 27 surgical case reports, again identified prostate as the leading primary (20.5%) and confirmed that cranial nerve palsies dominate the presentation (7). The anatomical basis for this distribution is Batson’s vertebral venous plexus, which allows tumour emboli from pelvic veins to bypass the pulmonary filter and seed the vertebrae, basiocciput and skull base directly (6). The axial, pulmonary and nodal pattern in our patient fits this mechanism. Mass lesions in compartments adjacent to the cavernous sinus, such as Meckel’s cave tumours, can produce overlapping but slightly different cranial nerve patterns; careful clinical localisation directs both imaging and surgical planning (17).

The severe hyponatraemia fitted a euvolaemic, SIAD-type picture. A serum sodium of 112 mEq/L combined with a spot urine sodium of 43 mEq/L indicated continued renal sodium excretion despite low plasma values. Together with normal thyroid, adrenal and renal function, no diuretic exposure, and clinical euvolaemia, this picture supported a diagnosis of SIAD as outlined in the 2014 European guideline (18). SIAD is the commonest cause of hyponatraemia in malignancy and arises from ectopic arginine-vasopressin release, pain, nausea, or cancer treatment (19). Management followed the same guideline: fluid restriction first, tolvaptan added when sodium failed to correct adequately.

Diagnosis relied on the combination of a grossly elevated PSA, the characteristic imaging pattern on multiparametric MRI (PIRADS 5 with extraprostatic extension), and tissue confirmation by transrectal ultrasound-guided biopsy.

Whether the rapid neurological decline and the systemic derangement were coincidental or part of a single process deserves comment. They were most likely not independent. The ophthalmoplegia, the anaemia of chronic disease, and the SIAD all emerged within the same admission, and each is a recognised consequence of a large, disseminated tumour burden rather than a separate disease. The clival deposit produced the cranial nerve signs directly; the anaemia reflected chronic inflammation, with extensive skeletal metastases likely contributing; and the SIAD was a paraneoplastic accompaniment of advanced malignancy. A high tumour burden declaring itself in several systems at once is a more economical explanation than three unrelated events. Most reports of clival or skull base prostate metastasis do not comment on the tempo of cranial nerve symptom progression; among the few that describe onset, the picture is one of rapid presentation, with Douglas reporting sudden-onset diplopia and Malloy sudden blurred vision as the first symptom (10, 11). Our patient likewise presented with sudden diplopia, and the ophthalmoplegia evolved over roughly three days, a comparably rapid course. We cannot prove a single mechanism from one case, but the convergence of three tumour-driven processes within the same week is most consistent with simultaneous multi-system involvement from widespread disease.

The most actionable message from this case is about opportunistic enquiry. Five months of poor urinary stream and hesitancy in an elderly man went unreported because he and his family considered these symptoms an inevitable part of ageing, a common pattern in rural Indian practice. The relationship between lower urinary tract symptoms (LUTS) and prostate cancer in older men is counterintuitive: roughly 70% of prostate cancers originate in the peripheral zone, anatomically distant from the urethra, while LUTS are generated by central and transitional zone enlargement that is almost always benign (20). LUTS are therefore a poor screening sensor for curable prostate cancer. Most older men with LUTS do not harbour cancer, and many men with cancer have no LUTS (20). In advanced disease, however, LUTS can reflect local extraprostatic invasion or bladder outlet obstruction from tumour bulk, as our patient’s PIRADS 5 lesion with extraprostatic extension illustrates. Despite the weak predictive value of LUTS for prostate cancer, their presence in an elderly man should still prompt PSA testing, because waiting for a more specific clinical sign typically means waiting until curative treatment is no longer possible. The 2024 EAU guidelines recommend risk-adapted PSA-based early detection in men aged 50 and above, with earlier testing in high-risk groups (21). Targeted enquiry about urinary symptoms and weight loss in older men with unexplained systemic findings remains the simplest route to earlier diagnosis. Underlying this individual encounter is a wider problem in low- and middle-income settings, where late diagnosis is the norm and more than half of men already have advanced disease when prostate cancer is found (4). The Lancet Commission identifies non-reporting of symptoms, low public awareness, and limited health literacy as central drivers, and recommends education of both the public and primary-care professionals, linked to community outreach, as the main route to shifting presentation from advanced to earlier-stage disease (4).

This report has two limitations. Paired plasma and urine osmolality, part of the formal diagnostic criteria for SIAD, were not measured (18). In addition, the patient declined androgen deprivation therapy in favour of alternative medicine and did not return, so we cannot report whether his cranial nerve deficits or survival followed the course seen in comparable cases.

## Conclusion

Clival metastasis producing acute cavernous sinus syndrome can be the first visible manifestation of otherwise silent disseminated prostate adenocarcinoma. A high index of suspicion, prompt imaging of the brain, and a low threshold for PSA testing in elderly men will catch a proportion of these patients earlier.

## Data Availability

The original contributions presented in the study are included in the article/**Supplementary Material**. Further inquiries can be directed to the corresponding author.
